# Clinical, physiological and cerebral effects of a remote adapted physical activity program in patients with schizophrenia

**DOI:** 10.1192/j.eurpsy.2023.132

**Published:** 2023-07-19

**Authors:** S. Dollfus, PEPSY group (E.Leroux,M.Trehout, L.Bigot, A.Turgis, E.Reboursiere, C.Vincent, R.DeFloers, R.Morello, O.Guillin, A.Herbinet, G.Quarck)

**Affiliations:** ^1^ Inserm U1237; ^2^CHU Caen, Caen, France

## Abstract

**Abstract: Background:**

Physical activity (PA) has emerged as an interesting adjuvant non-pharmacological intervention in patients with schizophrenia (SZPs). The vast majority of programs are face-to-face without considering the patients’ physiological capacities and their difficulty to achieve the programs. The aim of this study was to demonstrate the efficacy of PA on clinical variables and brain plasticity. Its originality was to adapt PA on the cardiorespiratory and physical capacities (APA) and to deliver PA remotely by a videoconference coach (e-APA).

**Methods:**

This longitudinal study included 35 SZPs (DSM-5) randomized either in an e-APA group or in a control group (health education training (e-HE)). Both programs were delivered in the same conditions, remotely via the web with a professional, for two 60-minute sessions per week during 16 weeks. Cardiorespiratory capacity measured by VO_2_max, clinical symptoms assessed with PANSS, BNSS and SNS, total hippocampus (HCP) volumes and their subfields, were evaluated in pre- (session 1) and post- interventions (session 2). High-resolution T_1_-weighted and two high-resolution T_2_w brain volumes were proceeded at session 1 and 2 (MRI 3-T, Philips). ANCOVAs were performed to determine intervention and/or diagnostic effects on relative variation (RV) of cardiorespiratory capacity, clinical symptoms and HCP volumes.

**Results:**

The retention rate of SZPs in the study was 88.6%. SZPs of e-APA group presented a greater RV of VO_2_max (+7.3%) compared to SPZs-HE (-3.9%) (p = 0.024). No significant effect of the e-APA compared to the e-HE was demonstrated regarding the RV of the clinical symptoms. However, between 1 and 2 sessions, total PANSS scores, positive and general PANSS sub-scores significantly decreased in both groups while total SNS and BNSS scores only decreased in e-APA group. Finally, a positive and greater RV of the left subiculum volume was observed in e-APA (+3.4%) compared to e-HE (-2.5%) (p = 0.0005).

**Conclusion:**

This study is the first one demonstrating the feasibility and acceptability of a remote APA program in SZPs with high participation rates. Our results show that e-APA induces brain plasticity reflected by an increase of HCP subfield volume and improves the cardiorespiratory capacity in SZPs. This study underlines that remote APA represents an innovative, original, safe and effective adjunctive therapeutic strategy in schizophrenia.

**Disclosure of Interest:**

None Declared

